# Naltrexone/Bupropion/Mirtazapine Triple Therapy for Treatment-Resistant Methamphetamine Use Disorder: A Case Series

**DOI:** 10.7759/cureus.102744

**Published:** 2026-01-31

**Authors:** Nima Shariatzadeh, Sidarth Wakhlu

**Affiliations:** 1 Long School of Medicine, University of Texas Health Science Center at San Antonio, San Antonio, USA; 2 Department of Psychiatry, University of Texas Southwestern Medical Center, Dallas, USA

**Keywords:** bupropion, methamphetamine abuse, methamphetamine use, mirtazapine, naltrexone, naltrexone-bupropion, stimulant abuse, stimulant treatment, stimulant use disorder

## Abstract

Stimulant use disorders (StUDs) remain a growing public health concern in the United States, with no FDA-approved pharmacological treatment available. While medications such as naltrexone (NTX), bupropion (BUP), and mirtazapine (MIRT) have shown modest benefits individually or in combination as BUP/NTX, no prior studies have examined their combined use as a triple therapy. Here, we present a case series of three patients with severe, treatment-resistant methamphetamine use disorder treated with a combination of NTX/BUP/MIRT for 18-24 months. Clinical outcomes such as methamphetamine use via urine drug screening, psychiatric comorbidity symptoms, and quality of life were monitored over time. All three patients experienced clinically meaningful reductions in methamphetamine use following initiation of triple therapy. Improvements were sustained over months and were accompanied by enhanced mood and improved psychosocial functioning. NTX/BUP/MIRT therapy was well-tolerated, with no major adverse events. These cases suggest that NTX/BUP/MIRT triple therapy may offer a promising pharmacologic strategy for patients with refractory StUDs. The complementary pharmacologic profiles of the three agents may produce synergistic benefits by targeting craving, withdrawal, mood instability, and insomnia. While encouraging, these findings require validation in controlled clinical trials.

## Introduction

Stimulant use disorder (StUD) is a condition characterized in the Diagnostic and Statistical Manual of Mental Disorders, Fifth Edition (DSM-5) by a problematic pattern of stimulant use (such as cocaine, amphetamines, or methamphetamine) leading to clinically significant impairment or distress [1]. StUDs continue to pose a significant public health challenge both in the United States and around the world. The United Nations Office on Drugs and Crime 2025 World Drug Report cited a record high number of methamphetamine seizures in 2023, accounting for nearly half of all synthetic drug seizures worldwide. Its use has increased most rapidly in the Middle East and North Africa, with continued hubs of use in North America and Southeast Asia [2]. According to the 2021-2023 National Survey on Drug Use and Health (NSDUH), 9.7 million Americans (3.4%) misused stimulants in 2023 [3]. Dubbed the “fourth wave” of the ongoing opioid epidemic in the United States [4], the concurrent rise in fentanyl/methamphetamine co-use is particularly alarming. Methamphetamine is a highly potent synthetic central nervous system (CNS) psychostimulant that rapidly crosses the blood-brain barrier due to its lipophilic structure, triggering massive dopamine release and producing intense euphoria, heightened alertness, and profound pleasurable effects [5]. Chronic use causes neurotoxicity, including dopaminergic terminal damage observable via positron emission tomography (PET) imaging, with reduced dopamine transporter density in methamphetamine users compared to controls [6]. Withdrawal following cessation is severe, featuring profound depression (often with suicidal ideation), intense anxiety, extreme fatigue, irritability, and persistent cravings lasting weeks to months. Additional highly toxic effects include cardiovascular damage, hyperthermia, psychosis, and extreme weight loss [7]. One notable complication of methamphetamine use involves intracerebral hemorrhage (ICH). Multiple studies have shown an increased association between methamphetamine use and ICH [8]; additionally, patients diagnosed with ICH who use methamphetamine tend to be younger at diagnosis [9]. Interestingly, the incidence of ICH may also serve as a temporal epidemiological indicator of increasingly widespread methamphetamine use and its complications. Indeed, one study found an increasing frequency of patients with ICH testing positive for methamphetamine in the Philippines, a country with the highest prevalence of methamphetamine use in Southeast Asia [10].

The complexity of methamphetamine addiction, compounded by frequent co-occurring mental disorders, presents additional challenges to treatment efforts [11]. Both UpToDate and the joint guidelines of the American Society of Addiction Medicine (ASAM)/American Association of Addiction Psychiatry (AAAP) conditionally recommend naltrexone(NTX)/bupropion (BUP) or mirtazapine (MIRT) as two options to be considered for pharmacological management of amphetamine-type stimulant disorders [12]. However, no studies to date have reported using all three compounds together as triple therapy. The data supporting BUP or NTX individually to treat amphetamine use are sparse. BUP, an atypical antidepressant with norepinephrine-dopamine reuptake inhibition (NDRI) and antagonism of certain nicotinic acetylcholine receptors, has shown some efficacy in treating both cocaine and methamphetamine use disorder [13]. A 2023 meta-analysis of placebo-controlled randomized clinical trials found that BUP was associated with reduced amphetamine-type stimulant cravings and use [14]. Conversely, NTX is a μ- and κ-opioid antagonist used to reduce cravings in alcohol and opioid use disorders [15]. NTX may also reduce stimulant cravings via reduced endorphin activity [16]. Some studies suggest NTX may also be efficacious as a standalone treatment for StUDs; one found NTX led to reduced amphetamine use and cravings in subjects with amphetamine dependence [17]. In another study, NTX reduced cue-induced craving and hedonic subjective effects after methamphetamine administration in subjects with methamphetamine use disorder [18]. Most recently, the ADAPT-2 trial tested a combination of NTX/BUP against placebo in a double-blinded fashion. This trial, the most carefully constructed to date, found a small but significant effect of NTX/BUP in reducing methamphetamine use [19].

Originally marketed as an atypical antidepressant, MIRT has also shown efficacy in the treatment of StUDs, especially methamphetamine use disorder. Its mechanism of action combines antagonism of various histaminergic, serotonin (5-HT), and α-adrenergic receptors, chiefly among these the H1, 5-HT2A, 5-HT2C, 5-HT3, and α2 family of receptors [20]. These actions lead to a downstream increase in monoamines, including dopamine, norepinephrine, and serotonin. Alterations of these monoamines in the mesolimbic system are postulated to reduce craving and withdrawal symptoms [21]. First discovered to attenuate signs of amphetamine withdrawal symptoms in a 2005 Thai study [22], subsequent randomized controlled trials established the efficacy of MIRT alone for methamphetamine use. A 2022 meta-analysis found a small but significant reduction in methamphetamine use for MIRT compared to placebo [23].

Modest effect sizes of BUP, NTX, BUP/NTX, and MIRT greatly limit their utility as a standalone treatment. To date, no work has examined the efficacy and tolerability of combining BUP, NTX, and MIRT as a unified treatment strategy for methamphetamine use disorder. This case series documents three patients with treatment-resistant methamphetamine use disorder who received a combination of NTX/BUP/MIRT triple therapy for 18-24 months and experienced significant improvement in their methamphetamine use and overall quality of life.

## Case presentation

Case 1

A 56-year-old woman with a prior medical history of pulmonary hypertension, hepatitis C, alcohol use, and chronic methamphetamine use was referred to our clinic for help with substance use and depression/anxiety. She reported methamphetamine use from the age of 15. Her last use was a few days before the appointment, and she reported using “one to two puffs” per day. She also reported significant alcohol use starting as a teenager and attendance of multiple inpatient rehabilitation programs for alcohol. She denied any psychiatric history but reported depressive symptoms (depressed mood, increased feelings of guilt and worthlessness, increased sleep, low motivation, and poor concentration) that she related to current methamphetamine-related legal problems. She also endorsed excessive worrying and anxiety during this time. The patient reported a history of auditory hallucinations for 21 years, starting a few months after the initiation of methamphetamine use. They consisted of an intermittent man's or woman's voice commenting on her actions; they were not command hallucinations. These voices were “dormant” during a prior period of incarceration for multiple years. Upon presentation to our clinic, she had been abstaining from alcohol use.

The patient was initially started on escitalopram, and NTX was titrated to 20 mg and 50 mg, respectively. After four weeks, she was started on risperidone to address psychotic symptoms concurrent with continued methamphetamine use. At a follow-up months later, she stated that escitalopram had little benefit and complained of insomnia. Escitalopram was discontinued and was replaced with extended-release BUP titrated to 300 mg; trazodone was added for insomnia. At follow-up six weeks later, the patient reported that the NTX/BUP combination had allowed her to further reduce methamphetamine use; her use declined from 0.5-1 gram daily to only once every two to three weeks. At this visit, she complained of morning-after grogginess with trazodone. Trazodone was replaced with MIRT 15 mg. At follow-up six weeks thereafter, the patient reported marked improvement in her insomnia and tolerated the MIRT well. She has cut back on her methamphetamine use “a lot” and has reported “one to two slips” of methamphetamine use lasting “just a day” in the past nine months. Reduction in methamphetamine use was confirmed both via urine drug testing and clinical impression. Since beginning triple therapy, urine drug screens were performed every four to six weeks for one year and every eight weeks thereafter. There was a 75% reduction in her urine drug testing positive for methamphetamine. She was negative for other illicit drug use or ethyl glucuronide (ETG). Her mood symptoms are now well-controlled, and her psychosis is markedly improved.

Case 2

A 42-year-old man with a prior history of human immunodeficiency virus (HIV), bipolar disorder, attention deficit hyperactivity disorder (ADHD), methamphetamine use, and alcohol use was referred to our clinic for continuation of psychiatric care. The patient reported struggling with ADHD and depression since his teenage years; methamphetamine and alcohol use began during this time. He previously attended multiple residential treatment programs to cease methamphetamine use, but was unable to remain abstinent for more than one month. At intake to our clinic, the patient reported smoking 2 grams of methamphetamine and drinking five to six 12-oz cans of beer daily. 

The patient was started on NTX 50 mg daily, reducing his methamphetamine use from every one to two days to every three to four days. Eight months later, extended-release BUP was initiated and titrated up to 450 mg. The addition led to further improvement, with the patient only using methamphetamine sporadically every three to four weeks. He continued this regimen for four years. During this time, the patient complained of insomnia, and MIRT was added to the regimen at a dose of 15 mg at bedtime, titrated to 30 mg. He reported marked improvement. He was able to completely stop methamphetamine six months later and now regularly attends both talk therapy and Narcotics Anonymous meetings. He has reported no recurrence of mood symptoms, and all subsequent urine drug screens were negative for methamphetamine, ETG, and other illicit drugs.

Case 3

A 47-year-old married man with a prior history of substance use and psychosis presented to our clinic to address his psychotic symptoms. He was brought by his wife, who reported that the patient used methamphetamine daily. He was paranoid and convinced his wife was having an extramarital affair; collateral information suggested he was experiencing stimulant-induced delusions. After inpatient psychiatric stabilization, the patient entered an inpatient residential treatment program for methamphetamine use.

During his hospitalization and residential treatment program, he was started on 300 mg BUP daily and 3 mg risperidone nightly. After discharge, the patient continued to struggle with methamphetamine use; he subsequently entered seven treatment programs over the next four years. He was able to abstain for a maximum of three to six months during this time. After his most recent discharge from a residential program, he was prescribed NTX titrated to 50 mg daily and hydroxyzine 50 mg for sleep. Following the addition of NTX, his methamphetamine use decreased substantially. He could now go several days without using methamphetamine. Due to inadequate response, hydroxyzine was replaced with MIRT 15 mg at bedtime, titrated up to 30 mg after a few weeks. At follow-up four weeks later, he reported no desire to use methamphetamine. The patient continues to tolerate the combination of NTX/BUP/MIRT and risperidone well. The patient has maintained sobriety for ten months, longer than any period during his time at our clinic. This period of sobriety was verified by the negativity of all drug tests for methamphetamine, ETG, or other illicit drugs. He is gainfully employed and lives with his wife and daughter.

## Discussion

Despite the chronicity of their stimulant use, as well as various psychiatric co-morbidities, all three patients experienced clinically meaningful reductions in methamphetamine use after initiation of NTX/BUP/MIRT triple therapy. These reductions were concomitant with clinical stabilization, improved mood, and enhanced quality of life. These findings suggest that in patients with failed attempts to reduce or eliminate methamphetamine use, NTX/BUP/MIRT may be a valuable pharmacological tool to treat methamphetamine use disorder while improving psychosocial functioning. 

The efficacy of this regimen may lie in synergistic effects between its components; theoretical understanding of individual agent pharmacology may yield clues as to their synergy. BUP’s NDRI effects may help mitigate post-withdrawal dysphoria and restore motivation by enhancing dopaminergic transmission, which is thought to be dysregulated in chronic stimulant use; these pharmacodynamics directly target withdrawal symptoms of fatigue and anhedonia while modulating cravings by re-establishing dopamine signaling equilibrium [24]. NTX’s antagonism of μ-opioid receptors may reduce cravings while simultaneously attenuating the rewarding effects of stimulants should use occur. Finally, MIRT’s antagonism of 5-HT2, 5-HT3, and norepinephrine-enhancing properties may further alleviate stimulant-withdrawal symptoms while also targeting post-withdrawal cravings, anxiety, and sleep disturbances [25]. Clinicians must still be mindful of deleterious side effects and when they may be dangerous. BUP is known to lower the seizure threshold and thus must be used cautiously in patients with a history of seizure disorders. In stimulant users, these can commonly occur from stimulant overdose or from possible comorbid conditions such as an eating disorder. While NTX has a minimal side effect profile, it may cause hepatotoxicity or precipitate opioid withdrawal in dependent patients and must be used cautiously in susceptible populations. Finally, MIRT may lead to skin reactions and serotonin syndrome when combined with other serotonergics. Patients should be educated on these risks and advised on the importance of avoiding recreational serotonergic drugs. Both BUP and MIRT should be used very cautiously in patients with a history of bipolar disorder, as they may precipitate mania. 

Triple therapy was able to both reduce stimulant use and also address psychiatric complaints in patients with various psychiatric comorbidities, including mood disorders, psychosis, and ADHD. Rather than exacerbating psychiatric illnesses, the regimen led to improved psychiatric functioning and was well-tolerated across all patients. Complementary side effect profiles of the three drugs may partially account for the tolerability: NTX and BUP are known to cause insomnia and appetite suppression [26]; conversely, MIRT can improve appetite and sleep [27]. The hypnotic effect of MIRT may also directly support abstinence, as insomnia is a frequent withdrawal symptom of stimulant use and may contribute to relapse [28]. These hypotheses are supported by the addition of MIRT, leading to further reductions in use and improvements in quality of life. By targeting stimulant addiction through independent and complementary mechanisms, triple-drug therapy was both efficacious and tolerable. 

This case series is limited by its small sample size (n=3), single-case design, and lack of controls, randomization, or blinding, which restrict generalizability, preclude causal inference, and increase risks of selection bias, observer bias, placebo effects, and confounding by unmeasured factors (e.g., psychosocial support, natural course). Safety and tolerability data are preliminary and insufficient to assess risks in larger or higher-risk populations. While promising, these findings are hypothesis-generating only; larger, controlled trials with objective endpoints and longer follow-up are needed to confirm efficacy and safety before clinical adoption.

## Conclusions

Taken together, these cases raise the prospect of NTX/BUP/MIRT triple therapy as an efficacious pharmacotherapy for StUDs in patients who have not responded to conventional therapies. Results must be interpreted cautiously due to the small sample size, lack of placebo control, and inherent biases of case series. Despite these limitations, our findings warrant the formal investigation of NTX/BUP/MIRT triple therapy through double-blind, randomized controlled trials. Future work should quantify the improved efficacy of triple therapy over its individual components and assess optimal dosing strategy, efficacy across various patient populations, and potential adverse effects. Given the complex medical and psychiatric presentations of people with stimulant use disorders, monitoring for drug-drug interactions and exacerbation of pre-existing conditions will be critical. Given the ongoing rise in stimulant use throughout the United States and lack of effective treatment options, NTX/BUP/MIRT triple therapy may represent an efficacious and accessible pharmacological regimen in the expanding fight against StUDs.
